# Assessment of Objective Response Rate by Investigator vs. Blinded Independent Central Review in Pivotal Trials of Oncology Drugs for Solid Tumor Indications

**DOI:** 10.3390/cancers17071096

**Published:** 2025-03-25

**Authors:** Marjorie E. Zettler

**Affiliations:** Proclinical Staffing Inc., Philadelphia, PA 19123, USA; marjoriezettler@gmail.com

**Keywords:** tumor response, objective response rate, efficacy, surrogate endpoint, drug development, clinical trial

## Abstract

**Background/Objective:** Objective response rate (ORR) is a surrogate endpoint frequently employed in early-phase clinical trials of anticancer agents for the treatment of solid tumors. Assessments of ORR by local investigators tend to be influenced by subjective factors, and blinded independent central review (BICR) is recommended by regulatory agencies in order to detect evaluation bias. The objective of this analysis was to compare BICR-assessed vs. investigator-assessed ORRs in pivotal trials of cancer drugs recently approved by the United States Food and Drug Administration (FDA) for solid tumor indications. **Methods:** The FDA’s Novel Drug Approvals reports were reviewed to identify cancer therapies approved for solid tumor indications between 1 January 2020 and 30 June 2024. Among therapies with ORR as a primary endpoint in pivotal trials, and for which both BICR- and investigator-assessed ORRs were available, a pooled analysis was conducted to compare these ORRs (using the Mantel–Haenszel method). A correlation analysis was also performed to evaluate the concordance between ORR assessments. **Results:** A total of 20 anticancer agents met the criteria for inclusion in this analysis, each supported by a single pivotal trial. Comparing BICR- and investigator-assessed ORRs in a pooled analysis did not identify any significant difference between the two assessments overall: OR = 0.98 (95% CI: 0.87–1.11), *p* = 0.75, and I^2^ = 0%. The correlation analysis also revealed a high level of concordance between BICR- and investigator-assessed ORRs, with r = 0.96 (*p* < 0.05). **Conclusions:** This study found no evidence of evaluation bias in the assessment of ORR among registrational trials supporting recent FDA approvals of anticancer agents for solid tumor indications.

## Simple Summary

The efficacy of cancer therapies for solid tumors can be determined by measuring tumor response in clinical trials. Previous studies have shown that investigator assessments of objective response rate (ORR) are prone to bias, detectable using a blinded independent central review (BICR) of radiological images. This study compared BICR- and investigator-assessed ORRs in pivotal trials of anticancer agents recently approved by the Food and Drug Administration for the treatment of solid tumors to evaluate the presence and extent of bias. No significant difference was found between the two ORR assessments. Using BICR remains an important precautionary measure to ensure treatment effect is accurately captured.

## 1. Introduction

Cancer is the second leading cause of death in the United States (US). In 2025, an estimated 618,120 people in the US will die from cancer, and there will be more than two million new cancer diagnoses [1]. Despite these sobering statistics, cancer mortality has declined in recent years, a trend attributed in part to the development of new therapies. As the number of novel anticancer agents in development has multiplied, surrogate endpoints have increasingly been adopted to measure treatment effect in clinical trials. Using these endpoints allows for the prediction of clinical benefit earlier, and with a smaller number of patients, than would be required for a clinical outcome like overall survival (often considered the gold standard of endpoints in oncology clinical trials). Among registrational studies of therapies for solid tumor indications, objective response rate (ORR), a measure of tumor bulk reduction based on radiologic image assessment using response evaluation criteria in solid tumors version 1.1 (RECIST v1.1) [2], is the most commonly utilized primary endpoint [3]. However, several studies have indicated that assessments of ORR by local investigators and by blinded independent central review (BICR) can be discordant, with local investigators more often reporting exaggerated tumor responses compared with BICR assessments [4,5]. Reasons for incongruous assessments may include differences in target lesion selection or identification of new lesions, variability in tumor measurement, or inconsistent interpretation of protocol-defined progression. Local investigators may also be more prone to influence as a result of their knowledge of the patient’s clinical status. This bias may be particularly apparent in open-label trials, where the assigned treatment is known to the investigator. To mitigate potential evaluation bias, regulatory guidance from both the US Food and Drug Administration (FDA) and European Medicines Agency (EMA) recommends (but does not require) that tumor response be assessed by BICR in registrational trials [6,7,8,9]. The FDA addresses this point in both the 2018 guidance Clinical Trial Endpoints for the Approval of Cancer Drugs and Biologics (“An independent endpoint review committee (IRC) can minimize bias in radiographic interpretation of the radiological findings and independent adjudication of assessments”) and the 2023 guidance Clinical Trial Considerations to Support Accelerated Approval of Oncology Therapeutics (“To reduce the potential to introduce bias and to mitigate variance in the assessment of response, blinded independent central review (BICR) of the response assessment should be performed”) [6,7]. The EMA’s 2023 Guideline on the Clinical Evaluation of Anticancer Medicinal Products (R6) states that “External independent review of tumour response is encouraged”, with Appendix 1 to the guideline adding that “…confidence in the quality of the trial will increase if the trial results from the BICR do not differ from the investigator assessments to any important degree” [8,9].

When both measurements are performed, the difference between them can be ascertained and quantified. For example, sonidegib (a hedgehog pathway inhibitor approved in 2015 for locally advanced basal cell carcinoma) demonstrated an ORR of 58% by BICR vs. 71% by investigator in the pivotal Phase 2 BOLT trial (NCT01327053) [10]. A discrepancy of 13% between these two different assessments may seem inconsequential; however, it is noteworthy that cancer therapies have been granted FDA approval based on even lower response rates (for example, the 2018 approval of the immune checkpoint inhibitor nivolumab for small cell lung cancer was supported by an ORR of 12% in the Phase 1/2 CheckMate-032 trial [NCT01928394]) [11]. When only one method of assessment is employed in pivotal clinical trials, no comparison can be made, and bias would not be detectable. This could conceivably mean the difference between the success and failure of a study: a trial utilizing only local investigator assessment, with its higher propensity for inflation, may report an ORR that appears to be clinically meaningful, but is in fact an overestimation. An accurate representation of treatment effect is critical to understanding the clinical benefit/risk profile of the drug and to inform decision-making for future development. An appraisal of the concordance between BICR- and investigator-assessments of ORR among contemporary clinical trials of cancer therapies could assist with interpreting the risk of bias associated with single-method ORR assessment.

This investigation aimed to compare BICR- and investigator-assessed ORRs among anticancer agents approved by the FDA for solid tumor indications between 1 January 2020 and 30 June 2024. The objectives were two-fold: first, to directly compare these two ORR assessment types and determine the magnitude of any difference between them, through a pooled analysis; and second, to calculate the concordance between these two assessments via a correlation analysis.

## 2. Methods

The FDA’s Novel Drug Approvals reports [12] were reviewed for cancer drug approvals for solid tumor indications occurring between 1 January 2020 and 30 June 2024. Supportive care drugs, diagnostic or contrast agents, and cell and gene therapies were excluded from this analysis.

For each identified solid tumor drug approval, drug approval packages and prescribing information were retrieved from the Drugs@FDA website [13], and the following characteristics were extracted: year of approval, indication, type of approval (accelerated vs. traditional), orphan drug designation status, and pivotal trial(s). The trials’ NCT numbers, phase, masking, and primary endpoint (BICR-assessed vs. investigator-assessed) were also extracted. For those trials with ORR as a primary endpoint, BICR-assessed and investigator-assessed ORR were collected. In cases where specific data points were not available from the drug approval package or label, the primary publication for the trial and the clinicaltrials.gov study record were reviewed for additional information.

For those trials with ORR as a primary endpoint and both BICR-assessed and investigator-assessed ORR available, a pooled analysis of study-level data was conducted using the Mantel–Haenszel method to compare ORRs by the two assessment methods. In some cases, more than one patient population was evaluated in the same trial (for example, treatment naïve subjects and pre-treated subjects). These were treated as independent comparisons (designated by the National Clinical Trial [NCT] number and year, followed by “a”, “b”, etc.); therefore, the total number of comparisons included in this analysis is greater than the number of trials.

The resulting odds ratio (OR; the ratio of BICR-assessed ORR to investigator-assessed ORR) indicated whether BICR or investigator evaluators were more optimistic with their ORR assessment. A *p* value less than 0.05 indicated significant differences between the two assessments. Analysis was conducted in Review Manager 5.4.

A correlation analysis was also undertaken to evaluate the concordance between the two ORR assessments. First, a test for normality was performed using the Shapiro–Wilk test. If normality was demonstrated, the Pearson correlation coefficient was calculated (otherwise, Spearman’s rank correlation coefficient was employed). A *p* value less than 0.05 indicated significant correlation between the two assessments. A scatterplot was generated for the correlation of BICR-assessed and investigator-assessed ORR using Microsoft Excel 2021.

All data analyzed in this study were collected from publicly available sources, and did not include identifiable health information for individual patients. Therefore, the protocol was not submitted for institutional review board approval.

## 3. Results

**Figure 1:**
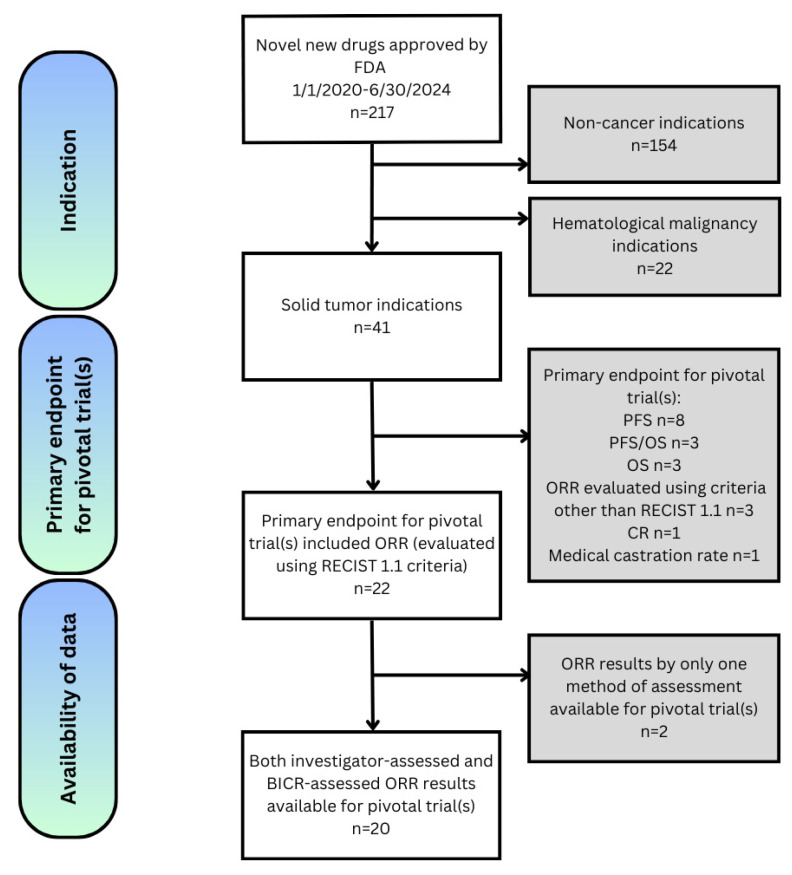
Flow chart of trial selection. CR = complete response; FDA = Food and Drug Administration; ORR = objective response rate; OS = overall survival; PFS = progression-free survival; RECIST= response evaluation criteria in solid tumors.

A total of 20 FDA approvals of cancer therapies indicated for solid tumors between 1 January 2020 and 30 June 2024 were identified for inclusion in this analysis (Figure 1).

The majority of these drugs were granted accelerated approval (18/20, 90.0%) and orphan drug designation (16/20, 80.0%). Each of these approvals was supported by a single pivotal trial. Characteristics of these trials are outlined in Table 1. For 17 of the 20 trials (85.0%), the primary endpoint specified BICR-assessed ORR, and the remaining 3 relied on investigator-assessed ORR. All 20 trials had an open-label design with no comparator; 19 of the 20 (95.0%) were Phase 1, 1/2, or 2.

**Figure 2:**
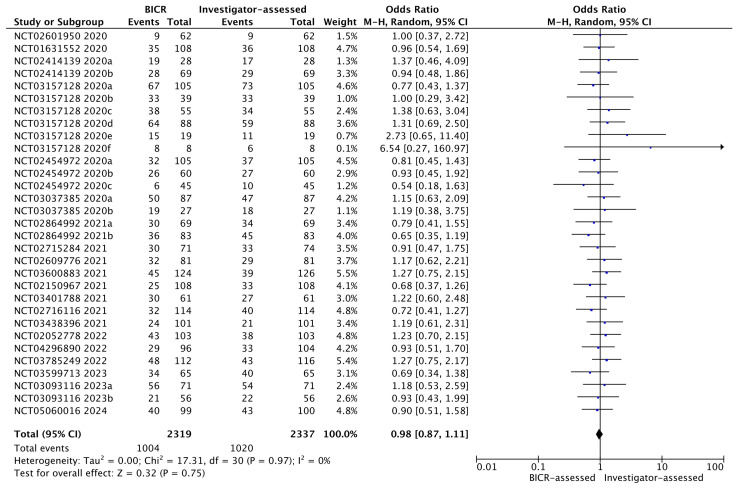
Forest plot for direct comparison of BICR-assessed and investigator-assessed ORRs. BICR = blinded independent central review; CI = confidence interval; M-H = Mantel–Haenszel; NCT = National Clinical Trial.

Comparisons between BICR- and investigator-assessed ORRs were made for each of the studies and subgroups (Figure 2). For 15 of the 31 pairs, the investigator-assessed ORR was numerically higher than the BICR-assessed ORR; for 14 of the 31 pairs the BICR-assessed ORR was numerically higher than the investigator-assessed ORR; and for the remaining 2 of the 31 pairs, the ORRs were identical. A pooled analysis comparing BICR- and investigator-assessed ORRs found no significant difference between the two assessments overall: OR = 0.98 (95% CI: 0.87–1.11), *p* = 0.75, and I^2^ = 0%. The ORs for individual studies are shown in Figure 2.

**Figure 3:**
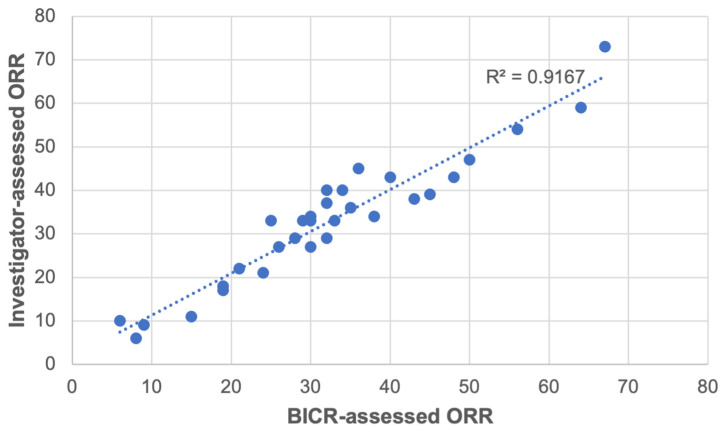
Scatterplot of correlation between BICR- and investigator-assessed ORRs. BICR = blinded independent central review.

Correlation analysis was undertaken as an estimation of the agreement between response rates as determined by the two methods of ORR evaluation. The results showed a positive correlation between BICR- and investigator-assessed ORRs. The association between values obtained by these two measures was very strong, with r = 0.96 (*p* < 0.05). Figure 3 illustrates the correlation of BICR-assessed and investigator-assessed ORRs.

## 4. Discussion

The present study is the first investigation comparing BICR and investigator assessments of response rate among contemporary pivotal oncology trials of novel anticancer agents where ORR (evaluated using RECIST 1.1 criteria) was the primary endpoint. These trials reflect circumstances where unreliable ORR estimates could have significant ramifications for the interpretation of the study results and subsequent market authorization and clinical use of the therapy. This analysis detected no evaluation bias between the two methods of ORR assessment in trials using tumor response to support FDA approvals of cancer drugs for solid tumor indications between 1 January 2020 and 30 June 2024. This finding aligns with most prior research on this topic conducted in the past decade, despite some previous analyses that focused exclusively on Phase 3 randomized controlled trials of therapies for solid tumor indications [14,15] and others that included trials of therapies for hematological malignancies or evaluations of response rates that used criteria other than RECIST 1.1 for response assessment [16,17]. In contrast, Dello Russo et al. [4,5] reported that local investigators significantly overestimated ORR compared with BICR in both Phase 2 and Phase 3 trials of anticancer agents. The two studies in question utilized a different measure than was used in the present work, a “discrepancy index” calculated for each trial as the ratio of investigator-assessed ORR to BICR-assessed ORR. In addition, the two analyses by Dello Russo et al. do not appear to have assigned weights to the trials and subgroups to ensure a proportionate contribution of each one toward the overall results. This is an essential step, as some smaller studies or subgroups with fewer events and larger confidence intervals may have a disproportionate impact on the overall point estimate. Using this methodology may have contributed to the discordant results compared to the present analysis and other studies in the literature.

None of the prior studies on this topic were limited to registrational trials with response rate as the primary endpoint. With the advent of precision medicine, there has been a shift towards smaller, earlier phase trials (often single-armed) supporting the accelerated approval of cancer therapies, many of which are indicated for rare cancers. Data indicate that two-thirds of accelerated approvals between 1992 and 2021 were for anticancer indications [18], and 65% of anticancer agents approved by the FDA between 2000 and 2022 were granted orphan drug designation [19]. In addition, of the 176 FDA approvals of cancer therapies between 2002 and 2021 based on single-arm trials, response rate was the endpoint used to support approval for 173 (98%) [20]. These factors can have an impact on the assessment of treatment effect: an analysis of FDA approvals of cancer drugs between 2000 and 2022 found that greater treatment effects for response rate were observed in pivotal Phase 1/2 studies compared with Phase 3 randomized controlled trials [21]. Larger treatment effects were also reported in smaller trials and trials without an active comparator [21]. In the present analysis, ORR was the most commonly used primary endpoint in registrational trials of cancer drugs approved by the FDA for solid tumor indications during the time period under study, all trials were open-label without a comparator, and all but one were early-phase. The majority of anticancer agents had orphan drug designation for the indication under study, and were granted accelerated approval.

Despite the frequency with which ORR is used to support accelerated approvals, it is an imperfect surrogate endpoint that may not reliably predict overall survival [22,23,24]. The disconnect can be due to factors such as treatment-related toxicity, which may result in death or treatment interruptions that prevent patients from deriving the full benefit of the therapy. Failure to adequately dose optimize an anticancer agent during development can contribute to avoidable toxicity and potentially affect survival outcomes [25]. One example of a targeted therapy with discordant ORR and overall survival findings is olaparib. The accelerated approval of olaparib on December 19, 2014 relied on investigator-assessed ORR in a single-arm Phase 2 study (NCT01078662) of 137 patients with germline BRCA mutation-associated ovarian cancer [26]. However, the confirmatory Phase 3 SOLO-3 trial [27] of 266 patients randomized 2:1 to olaparib or physician’s choice of single-agent nonplatinum chemotherapy demonstrated a negative impact of olaparib treatment on overall survival, and the drug was later voluntarily withdrawn from the market [28]. Cases such as this underscore the need for the timely completion of confirmatory trials to minimize the exposure of patients to cancer drugs that do not confer meaningful clinical benefit. To that end, the FDA has issued several new guidance documents, including Clinical Trial Considerations to Support Accelerated Approval of Oncology Therapeutics in March 2023 and Expedited Program for Serious Conditions—Accelerated Approval of Drugs and Biologics in December 2024, which highlight the requirement for post-approval confirmatory trials to be underway at the time of accelerated approval [7,29]. The FDA’s authority to require confirmatory trials to already be enrolling at the time of accelerated approval has already been exercised. In 2024, the FDA issued complete response letters to the sponsor of odronextamab, in development for the treatment of relapsed/refractory follicular lymphoma and diffuse large B-cell lymphoma, because enrollment in the confirmatory portions of the trials had not yet begun [30]. A new draft guidance was published by the FDA in early 2025 to define for sponsors when a confirmatory trial is considered to be underway [31]. In addition to these efforts to mitigate risks associated with the use of existing surrogate endpoints to support oncology drug approvals, the FDA also launched Project Endpoint in 2023, focused on the development of novel, early endpoints in oncology drug development [32]. Advancing this initiative represents a step towards prioritizing the identification of measures that may better predict clinical benefit than surrogate endpoints currently in use.

Implementing BICR for a clinical trial adds considerable complexity, as well as administrative and financial burdens. This could prove challenging for smaller biotech companies with limited resources at a time when a growing number of FDA approvals are sponsored by biotech companies [33]. It can also create operational issues due to the asynchronous nature of the BICR assessment: for example, when a local investigator determines that a patient’s disease has progressed, they may decide to end the experimental treatment and start a new therapy immediately. The BICR assessment may subsequently determine that the disease has not progressed; however, the patient has already been removed from the study. Real-time BICR could alleviate this problem, but such an approach would be logistically impractical. The use of BICR can thus complicate clinical trials, and it may not be worthwhile in all scenarios. One strategy could be to reserve BICR assessment for trials most at risk of evaluation bias. This could include trials of anticancer agents that have demonstrated only modest improvements in ORR, open-label trials, or randomized double-blind trials where one therapy has a unique toxicity profile, or where the cancer under study is characterized by specific, easily identifiable symptoms. An alternative to BICR assessment of all radiographic images in a trial could be an audit, whereby only a sample of images is evaluated by BICR. The BICR assessment could then serve as a sensitivity analysis compared to the investigator assessment. Finally, even if BICR is not performed during the trial (or is performed for a fraction of the images, as an audit), archiving all radiographic images would be a prudent course of action, allowing for a full BICR assessment at a later time if deemed necessary.

A limitation of this study is that all of the included trials supported drug approvals, and therefore had been successful in meeting their primary endpoint. The inclusion of trials that were intended to support registration but failed to meet their primary endpoint could be more instructive with respect to how incongruent BICR- and investigator-assessed ORRs impact drug development and regulatory decision-making. For example, a recent press release reported the results of the Phase 2 KEYNOTE-695 trial (NCT03132675), which evaluated TAVO™-EP (an interleukin 12 encoding plasmid delivered by intratumoral electroporation) in combination with pembrolizumab for the treatment of patients with unresectable or metastatic melanoma [34]. The primary endpoint of this open-label, single-arm trial was BICR-assessed ORR, and a clinically meaningful ORR was pre-specified in the protocol as at least 17%. The trial failed to meet this bar, as the BICR-assessed ORR was only 10.2%. However, the investigator-assessed ORR was 18.8%, which would have met the threshold had investigator-assessed ORR been selected as the primary endpoint.

## 5. Conclusions

Is BICR necessary for the evaluation of ORR in registrational trials? The results of this analysis showed no difference between BICR and local investigator assessments. However, in cases where the risk of bias in ORR assessment is higher, BICR represents a useful safeguard to confirm the validity of the result.

## Figures and Tables

**Table 1:** Characteristics of included trials.

NCT #	Trial	Phase	Masking	Investigational Drug	Comparator	Indication
NCT03037385	ARROW	1/2	open-label	pralsetinib	none	NSCLC
NCT02454972	PM1183-B-005-14	2	open-label	lurbinectedin	none	SCLC
NCT03157128	LIBRETTO-001	1/2	open-label	selpercatinib	none	NSCLC, thyroid cancer
NCT02414139	GEOMETRY mono-1	2	open-label	capmatinib	none	NSCLC
NCT01631552	IMMU-132-01	1/2	open-label	sacituzumab govitecan-hziy	none	breast cancer
NCT02601950	EZH-202	2	open-label	tazemetostat	none	epithelioid sarcoma
NCT03438396	innovaTV 204	2	open-label	tisotumab vedotin-tftv	none	cervical cancer
NCT02716116	AP32788-15-101	1/2	open-label	mobocertinib	none	NSCLC
NCT03401788	004	2	open-label	belzutifan	none	von Hippel-Lindau disease
NCT02150967	CBGJ398X2204	2	open-label	infigratinib	none	cholangiocarcinoma
NCT03600883	CodeBreaK 100	1/2	open-label	sotorasib	none	NSCLC
NCT02609776	CHRYSALIS	1	open-label	amivantamab-vmjw	none	NSCLC
NCT02715284	GARNET	1	open-label	dostarlimab-gxly	none	endometrial cancer
NCT02864992	VISION	2	open-label	tepotinib	none	NSCLC
NCT03785249	KRYSTAL-1	1/2	open-label	adagrasib	none	NSCLC
NCT04296890	0417	3	open-label	mirvetuximab soravtansine-gynx	none	ovarian cancer
NCT02052778	TAS-120-101	1/2	open-label	futibatinib	none	cholangiocarcinoma
NCT03093116	TRIDENT-1	1/2	open-label	repotrectinib	none	NSCLC
NCT03599713	PODIUM-201	2	open-label	retifanlimab-dlwr	none	Merkel cell carcinoma
NCT05060016	DeLLphi-301	2	open-label	tarlatamab	none	SCLC

NCT = National Clinical Trial; NSCLC = non-small cell lung cancer; SCLC = small cell lung cancer.

## Data Availability

Dataset available on request from the author.
